# Revisiting the Crystal Structure of Metal–Organic Framework UTSA-16 for Chemical Consistency

**DOI:** 10.1021/acsorginorgau.6c00018

**Published:** 2026-04-30

**Authors:** Giulio Bresciani, Marco Taddei

**Affiliations:** † Department of Chemistry and Industrial Chemistry, INSTM Research Unit, 9310 University of Pisa, Via G. Moruzzi 13, 56124 Pisa, Italy; ‡ Centro per l’Integrazione della Strumentazione scientifica dell’Università di Pisa (C.I.S.U.P.), University of Pisa, 56124 Pisa, Italy

**Keywords:** metal−organic frameworks, cobalt, potassium, crystallography, single crystal X-ray diffraction

## Abstract

The metal–organic framework UTSA-16, based on K^+^, Co^2+^, and citrate as the organic linker and first reported in 2005, is one of the most attractive candidates as a solid adsorbent for postcombustion CO_2_ capture. Here, we revisit the crystal structure of UTSA-16 on the basis of clear evidence that the amount of potassium has been systematically underestimated in previous literature reports dealing with crystallographic characterization. By combining CHN elemental analysis, inductively coupled plasma optical emission spectroscopy, and thermogravimetric analysis, we find that the actual chemical formula for UTSA-16 is K_2_Co_3_(cit)_2_·8H_2_O, with a K/Co ratio twice as large as the one assumed in previous literature. Single crystal X-ray diffraction analysis leads to identifying two extraframework positions occupied by previously overlooked K^+^ ions, which are likely to play a key role in the adsorption behavior of UTSA-16.

## Graphical abstract

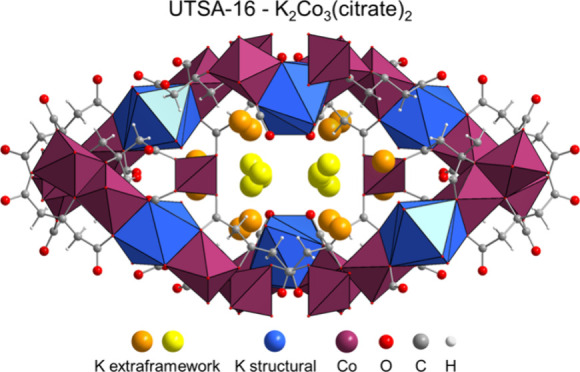

Metal–organic frameworks (MOFs) have enjoyed an ever-growing success over the last three decades, culminated with the award of the 2025 Nobel Prize in Chemistry to Richard Robson, Susumu Kitagawa and Omar Yaghi.^1–3^ A big part in this success has been played by the crystalline nature of MOFs, which allows to achieve a detailed, molecular-level description of their structure, thus enabling the establishment of structure–property relationships that, in turn, guide the rational design of new materials with the desired function.^4–6^ Such detailed description is not only limited to the framework itself, as the location of guest species that occupy the pores may also be determined, helping to understand the fundamental interactions that govern adsorption phenomena.^7–11^

Adsorption-based applications of MOFs are, in fact, the most intensely investigated, encompassing separation, storage and sensing of both gases and vapors, and the removal of both metal ions and organic pollutants from wastewater.^12–16^ Among these, CO_2_ capture is perhaps the most relevant, and many MOFs have been evaluated as solid sorbents, identifying several candidates that can outperform the benchmark zeolite 13 X.^17–19^ UTSA-16, a MOF based on K^+^, Co^2+^/Zn^2+^ and citric acid (H_4_cit), has garnered special attention because it is cost-effective, can be conveniently synthesized in water/ethanol medium, and displays excellent performance for postcombustion CO_2_ capture.^20–30^

The crystal structure of UTSA-16­(Co) was first solved from single crystal X-ray diffraction (SCXRD) data and reported in 2005 by Xiang et al.^31^ The MOF was formulated as [KCo_3_(Hcit)­(cit)­(H_2_O)_2_]·8H_2_O (CSD refcode RAZXIA) and was found to display a crystal structure with body-centered tetragonal symmetry, built from the connection of cubane-like tetranuclear clusters of octahedrally coordinated Co^2+^ and isolated tetrahedral Co^2+^ ions through the carboxylate and alkoxide groups of citrate linkers into a 3D (3,6)-connected anatase-type net with channels running along the *a* and *b* axis directions (Figure 1). K^+^ ions are located in special positions, surrounded by six O atoms from citrate and two coordinated water molecules. The location of the proton on the Hcit^3–^ linker was not determined.

**Figure 1:**
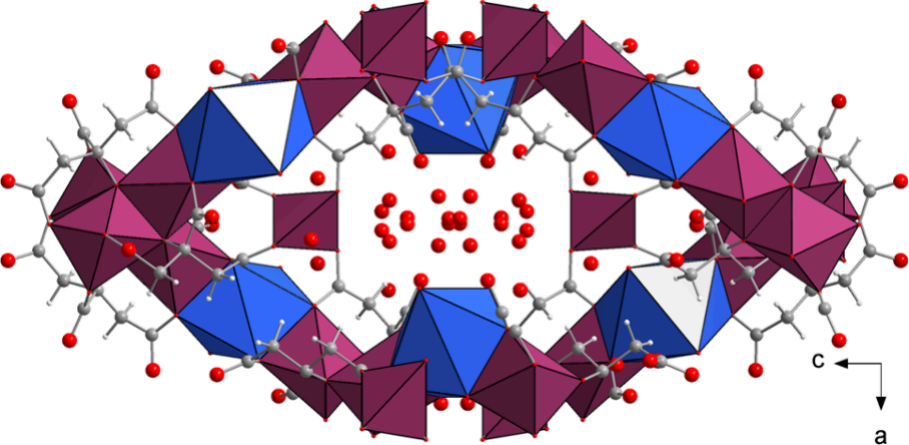
Polyhedral model of the crystal structure of UTSA-16­(Co) deposited in the CSD with refcode RAZXIA viewed along the *b* axis. Color code: Co, plum; K, blue; C, gray; O, red; H white.

A subsequent work, published in 2012,^20^ established the potential of UTSA-16 as a solid sorbent for CO_2_ capture. In this work, a structural model of the activated MOF loaded with CO_2_ was proposed based on neutron powder diffraction data. The authors sought no specific chemical information and assumed the same K/Co ratio of 1:3 of the original report, claiming that, upon activation, the two K-coordinated water molecules were retained in the MOF and served as the main adsorption sites for CO_2_, proposing the formula KCo_3_(Hcit)­(cit)·2H_2_O·0.89CO_2_ (CSD refcode GEBCAT). The proton on the citrate linker was located on the noncoordinated carboxylic O atom (O6) exposed inside the pores. Parsons and co-workers^32^ later reported a high-pressure SCXRD study, observing that UTSA-16 displays negative linear compressibility. In their structural analysis, the authors assumed the same formula proposed in the original paper by Xiang et al. (CSD refcodes RAZXIA01–02–03–04). The most recent crystal structures available in the CSD refer to UTSA-16 samples containing either a mixture of Co^2+^ and Zn^2+^ or just Zn^2+^ (CSD refcodes SUPKIA and SUPKOG, respectively).^26^ In this case, the authors still assumed a K/M^2+^ (where M^2+^ is Co^2+^ and/or Zn^2+^) ratio of 1:3 and only modeled the water molecules coordinated to K, using the SQUEEZE function in Platon^33^ to model the electron density in the pores.

A different chemical composition of UTSA-16 was proposed by Masala et al. in 2016,^34,35^ who found a K/Co ratio of 1:1.34 by energy dispersive X-ray spectroscopy, and that no water was retained in the MOF upon activation, as revealed by infrared (IR) spectroscopy. These observations led to propose the formula K_2_Co_3_(cit)_2_ for the activated MOF. The presence of two types of accessible Lewis acidic K^+^ sites was ascertained by *in situ* IR using H_2_, CO and CO_2_ as probes, suggesting that these are, in fact, the main adsorption sites for small molecules. No structural analysis was performed in these studies, and the additional K^+^ was only assumed to be located at the center of the pore.

Given the contrasting evidence presented in the literature regarding the chemical composition of UTSA-16, in this work we propose a revision of its crystal structure based on accurate chemical information. The importance of determining the chemical formula of MOFs was recently addressed by De Roo and co-workers,^36^ who proposed a general methodology to determine the experimental minimal formula of MOFs based on combined analytical techniques, highlighting the frequent discrepancies between crystallographic models and actual composition. Recent efforts toward standardized reporting of MOF composition and preparation, such as the Material Preparation Information File (MPIF), further highlight the importance of accurate chemical information.^37^

Single crystals of UTSA-16 were prepared in the same synthetic conditions used in the original paper by Xiang et al.,^31^ that is, by combining Co­(OOCCH_3_)_2_·4H_2_O, H_4_cit·H_2_O and KOH in a 1:1:3 molar ratio in mixed H_2_O/ethanol (50:50 *v*/*v*) medium and heating to 120 °C for 48 h (Figure S1). The crystals were ground and a powder X-ray diffraction (PXRD) pattern was collected to confirm phase purity (Figure S2).

By combining CHN analysis and inductively coupled plasma optical emission spectroscopy (ICP-OES) on the as-prepared MOF, we determined the following elemental contents: C, 18.7%; H, 2.9%; Co, 22.3%; K, 9.0%. Based on the KCo_3_(Hcit)­(cit)·10H_2_O formula proposed by Xiang et al.^31^ (formula weight = 773 g mol^–1^) the following contents are calculated: C, 18.6%; H, 3.8%; Co, 22.9%; K, 5.1%. Compared to the experimental values, the C and Co contents match well, however, the H content is overestimated and the K content appears significantly underestimated. On the other hand, the formula K_2_Co_3_(cit)_2_·8H_2_O (formula weight = 775 g mol^–1^), based on what proposed by Masala et al.,^34^ leads to calculate the following elemental contents: C, 18.6%; H, 3.1%; Co, 22.8%; K, 10.1%. The H and K contents are much closer to the experimentally determined ones.

Thermogravimetric analysis (TGA) conducted in oxidative environment (Figure 2a) displays a first step of mass loss taking place below 150 °C and accounting for 18.0% of the initial mass, caused by the desorption of water from the pores. Based on the formula KCo_3_(Hcit)­(cit)·10H_2_O, the complete release of 10 molecules of water per formula unit would account for 23.0% of the initial mass. Xiang et al.^31^ observed a similar mass loss, and attributed it to the release of 8 molecules of water per formula unit, accounting for 18.0% of the initial mass, while two molecules were assumed be retained in the coordination sphere of K. The loss of eight water molecules per formula unit according to the formula K_2_Co_3_(cit)_2_·8H_2_O accounts for 18.6% of the initial mass, in very good agreement with the experimental value.

**Figure 2:**
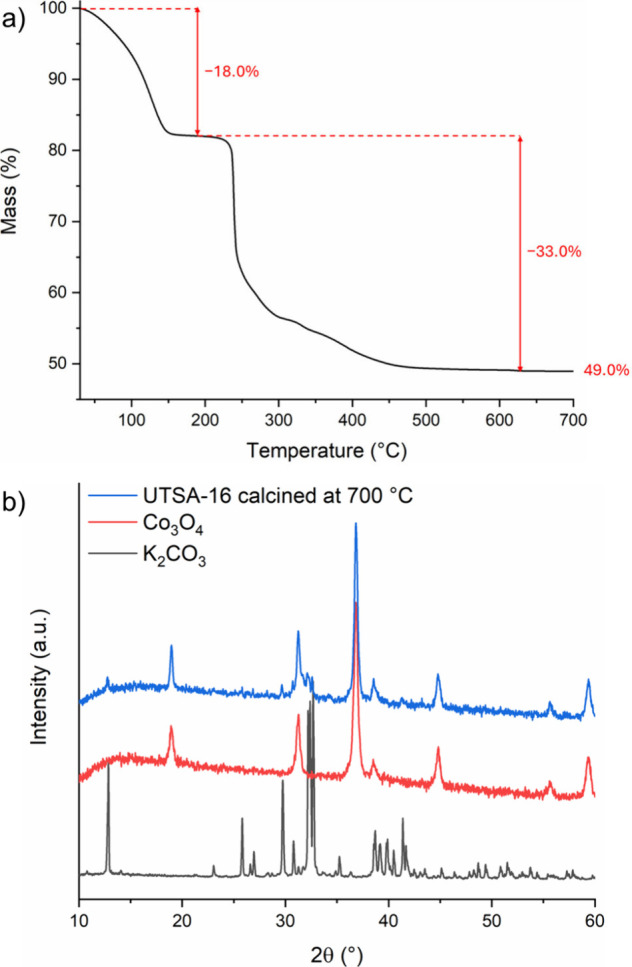
Thermogravimetric curve of UTSA-16­(Co) (a) and PXRD pattern of the residue after calcination at 700 °C (blue) compared with the patterns of Co_3_O_4_ (red) and K_2_CO_3_ (gray) (b).

The second mass loss (33.0%) takes place above 200 °C and leads to a residue accounting for 49.0% of the initial mass. We calcined UTSA-16 at 700 °C for 4 h and collected a PXRD pattern of the residual solid, finding that it is composed of a mixture of Co_3_O_4_ and K_2_CO_3_ (Figure 2b). If the formula KCo_3_(Hcit)­(cit)·10H_2_O is assumed for the MOF, the residue should consist of 0.5 equiv of K_2_CO_3_ (FW = 138.2 g mol^–1^) and 1 equiv of Co_3_O_4_ (FW = 240.8 g mol^–1^), which would account for 40.0% of the initial mass, a value much lower than the experimentally observed one. Based on the K_2_Co_3_(cit)_2_·8H_2_O formula, the residue is a 1:1 mixture of Co_3_O_4_ and K_2_CO_3_, accounting for 48.9% of the initial mass, in excellent agreement with the experimental value.

Having confirmed the formula K_2_Co_3_(cit)_2_·8H_2_O for as-synthesized UTSA-16, we performed SCXRD analysis to identify the location of the additional K^+^ ions not accounted for in any crystal structure present in the CSD for UTSA-16. To facilitate the identification of peaks of electron density attributable to K^+^, we loaded a crystal inside a capillary and gently heated it to 80 °C for 6 h under vacuum to remove the adsorbed water molecules. The capillary was then sealed under inert atmosphere to avoid water readsorption, and data collection was performed at – 173 °C. A body-centered tetragonal unit cell (space group *I*–42*d*) with lattice parameters *a* = 13.0209(3) Å, *c* = 30.016(1) Å, volume = 5089.0(3) Å^3^ was found, in agreement with the previously reported structures.

The bulk of the crystal structure, that is, two crystallographically independent Co atoms (one in octahedral coordination sitting in a general position and one in tetrahedral geometry sitting in a 8c special position), one structural K atom (sitting in a 8d special position) and the organic linker are in agreement with those of the previously reported structure (Figures S3–6). By analyzing the residual electron density in the pores, however, we identified two sites for extraframework K^+^ cations, both located in general positions (Figure 3a). In both cases, K was found to be disordered over two positions. Since the additional K^+^ cations identified here are located at extraframework positions within the pores and display partial occupancy, they do not alter the framework connectivity. Accordingly, the overall crystal packing and the underlying topology of UTSA-16 remain unchanged with respect to previously reported structural models.^20,26,31,32^

**Figure 3:**
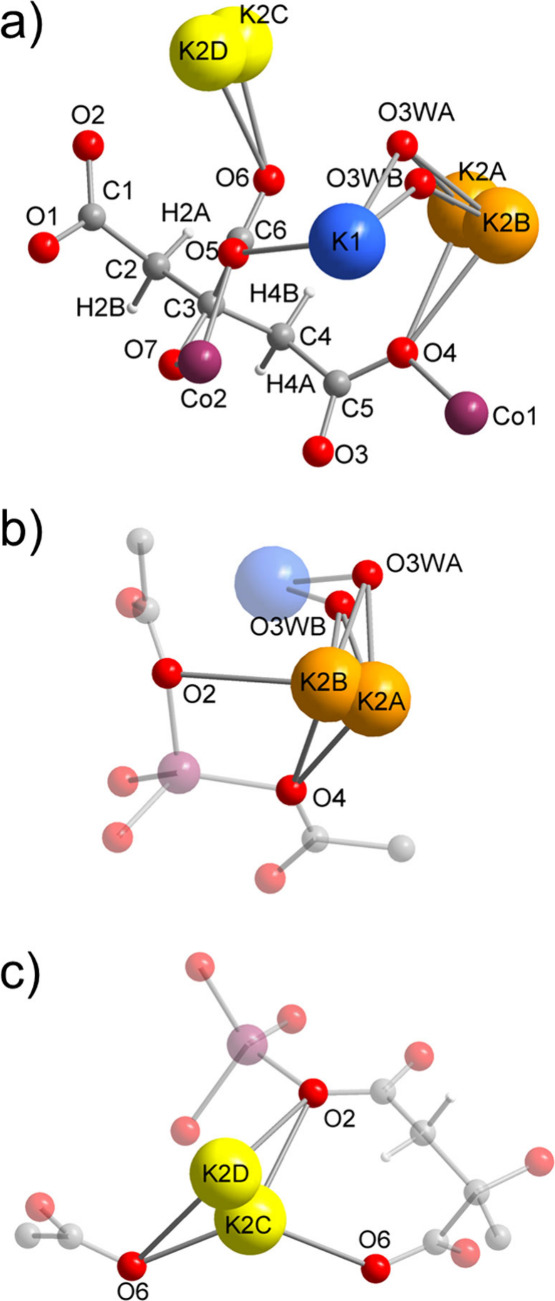
Asymmetric unit of UTSA-16­(Co) (a) environment of K2A-B (b) and environment of K2C–D (c). Atoms not directly interacting with K are shaded in (b) and (c) for the sake of clarity. Color code: Co, plum; K1, blue; K2A-B, orange; K2C–D, yellow; C, gray; O, red; H white.

All the disordered potassium atoms (K2A-D) were initially refined with free occupancy parameters. The sum of the resulting occupancies was close to what expected for the K_2_Co_3_(cit)_2_ stoichiometry, and was subsequently restrained to 0.5 to match the experimentally determined stoichiometry. The following individual values were found at the end of the refinement: K2A, 0.15(1); K2B, 0.05(1); K2C, 0.188(5); K2D, 0.113(6). In addition, we found residual electron density in approximately the same location as the K-coordinated water molecule (O3W) in RAZXIA.^31^ We attribute this residual electron density to the incomplete removal from the crystal of water molecules coordinated to K1 and K2A-B.

To investigate the chemical environment of the extraframework K species, a 3 Å threshold was set to determine contacts with O atoms. K2A and K2B are located in the proximity of two carboxylate oxygens (O2 and O4) already engaged in coordination with tetrahedral Co1 atoms and of OW3A-B (Figure 3b). K2C and K2D are located in the proximity of the noncoordinated carboxylate oxygen O6 and, again, O2, which is already engaged in coordination with tetrahedral Co1 (Figure 3c). It is interesting to note that the positions of the electron density peaks that we assign to K atoms here are reminiscent of those that were originally assigned to water molecules by Xiang et al.^31^ (Figures S3–6). This incorrect assignment can be explained based on the similar electron density associated with a partially occupied K^+^ (18 electrons) and a water molecule (10 electrons), underscoring, once more, the importance of the knowledge of the sample’s chemical composition. The higher sum of occupancies of K2C–D (0.30) than K2A-B (0.20) may be explained by noticing that K2C–D interact with two crystallographically equivalent O6 atoms that are not engaged in other coordination bonds and can serve as stronger donors than O4, O2 and O3WA-B. It is reasonable to expect that these strongly undercoordinated extraframework K^+^ ions, well exposed within the channels, can serve as additional adsorption sites for Lewis basic species, such as water and CO_2_.

In conclusion, we have presented a revised crystal structure for UTSA-16­(Co), one of the benchmark MOFs for CO_2_ capture applications. Based on accurate information on the chemical composition, we confirmed that the actual K/Co ratio is 2:3 and identified two extraframework sites for K^+^ ions that had been overlooked in the literature for more than 20 years. Given the potential role played by such species in the interaction with guests, we propose that our structural model becomes the reference for any consideration regarding the adsorption behavior of UTSA-16 from now on. We also wish to stress the importance of gathering direct evidence of the chemical composition of MOFs (or any other chemical compound) to support structural information, avoiding to rely solely on SCXRD data as a proxy for chemical composition. This approach is consistent with recent work by De Roo and co-workers,^36^ who recommend to determine the experimental minimal formula of MOFs through combined analytical techniques. In the present study, we extend this philosophy by directly integrating CHN elemental analysis, ICP-OES and TGA (supported with PXRD analysis of the calcined residue) with SCXRD, enabling the identification of previously overlooked extraframework species in UTSA-16.

## Supplementary Material



## Data Availability

The data underlying this study are available in the published article, in its Supporting Information, and openly available in Zenodo at 10.5281/zenodo.19549169.

## Notes

Experimental section, PXRD patterns and additional structural figures (PDF)
