# Supplementary material for: Revisiting the Crystal Structure of Metal–Organic Framework UTSA-16 for Chemical Consistency
Source: ACS Org Inorg Au. 2026 Apr 30;6(3):277–81. doi: 10.1021/acsorginorgau.6c00018 (PMC13237601; doi:10.1021/acsorginorgau.6c00018)
Supplement: Supplementary file 1 [file gg6c00018_si_001.pdf]

Revisiting the Crystal Structure of Metal-Organic Framework UTSA-16 for Chemical Consistency

Giulio Bresciani[1,2,*] and Marco Taddei[1,2,*]

[1]*Department of Chemistry and Industrial Chemistry, INSTM Research Unit, University of Pisa, Via G. Moruzzi 13, 56124, Pisa, Italy. Email:* giulio.bresciani@unipi.it marco.taddei@unipi.it

[b] *Centro per l'Integrazione della Strumentazione scientifica dell'Università di Pisa (C.I.S.U.P.), University of Pisa, 56124 Pisa, Italy*

SUPPORTING INFORMATION

Experimental

Materials and methods.

$Co(CH_3COO)_2{\cdot}4H_2O$ (*Carlo Erba*), citric acid monohydrate (*J.T. Baker*), KOH (*Sigma Aldrich*) and EtOH (*Carlo Erba*) were used as received without further purification.

Single crystals of UTSA-16 were obtained following the procedure reported in the original paper.[1] $Co(CH_3COO)_2{\cdot}4H_2O$ (249 mg, 1.00 mmol), citric acid monohydrate (210 mg, 1.00 mmol) and KOH (169 mg, 3.01 mmol) were placed in a 60 mL Carius Tube equipped with a rotaflow stopcock. The mixture of solids was suspended in 5 mL of a H_2O/EtOH mixture (1:1 *v*/*v*), heated to 120 °C for 48 hours and then cooled to room temperature over six hours. The prismatic purple crystals were recovered and washed with 3 mL of EtOH and dried in air for 18 hours. Yield: 189 mg (73.5%, based on Co).

Powder X-ray diffraction (PXRD) patterns were collected with a Rigaku MiniFlex diffractometer using Cu Kα radiation (1.54056 Å). The X-ray tube was operated at a voltage of 40 kV and a current of 15 mA. The XRF reduction function was employed to limit the contribution of fluorescence due to Co. The pattern of as-synthesized UTSA-16 was collected in the in the 3-40° 2θ range with a 0.02° step size, whereas the pattern of the calcinated UTSA-16 was collected in the 10-60° 2θ range with a 0.025° step size.

CHN elemental analysis was performed on a Vario MICRO cube instrument (Elementar). About 2 mg of sample was used.

Inductively Coupled Plasma-Optical Emission Spectrometry (ICP-OES) analysis was performed on a Thermo Scientific iCAP 7000 Series instrument. A multielement standard solution was employed (10 ppm, 5 ppm, 1 ppm, 0.5 ppm, 0.1 ppm). For sample preparation, 25 mg of the solid was accurately weighed and digested in 100.0 mL of a 3wt% HNO_3 solution. 2.0 mL of the resulting solution was diluted with 3% HNO_3 to a final volume of 20.0 mL.

Thermogravimetric analysis (TGA) was performed with a Perkin Elmer Pyris instrument using a heating rate of 10 °C min^{-1} in the temperature range of 30–700 °C under air flow (20 mL min^{-1}). 10.0158 mg of sample was used.

Single-crystal X-ray diffraction (SCXRD) data were collected on a Bruker D8 Venture diffractometer equipped with a Mo Kα microfocus source (λ = 0.71073 Å) and a Photon II 2D detector. Crystals were mounted in a glass capillary of appropriate size, heated (80 °C) under dynamic vacuum (ca 0.1 mbar) for 6 hours and then sealed under nitrogen atmosphere. Unit-cell determination and initial refinement were carried out using APEX4.[2] Data integration and reduction were performed with SAINT[3] and XPREP,[4] and absorption corrections were applied using SADABS.[5] Structures were solved via intrinsic phasing with ShelXT[6] and refined by full-matrix least-squares methods. All non-hydrogen atoms were refined anisotropically. Hydrogen atoms were placed in calculated positions and refined using a riding model. The potassium atoms K2A-D, disordered over multiple sites, were initially refined with free occupancy parameters (FVARs). The sum of the resulting occupancies was close to the $K_2Co_3(cit)_2$ stoichiometry, and was subsequently fixed to match this composition, in agreement with compositional analysis. Key experimental parameters are summarized in Table S1.

Crystallographic data have been deposited at the Cambridge Crystallographic Data Centre under the deposition number 2530260 and can be obtained free of charge via www.ccdc.cam.ac.uk.

Table 1. Crystal data and measurement details for **UTSA-16**

**Compound**	**UTSA-16**
CCDC ID	2530260
Formula	$C_{12}H_8Co_3K_2O_{15}$
FW, g mol^{-1}	647.17
T, K	100(2)
λ, Å	0.71073
Crystal system	tetragonal
Space group	*I*–42d (122)
a, Å	13.0209(3)
c, Å	30.016(1)
Cell Volume, Å^3	5089.0(3)
Z	8
D_c, g cm^{-3}	1.689
m,mm^{-1}	2.32
F(000)	2552
Crystal size, mm	0.102x0.075x0.050
2θ range,°	4.13 to 69.99
Reflections collected	68396
Independent reflections	5576 [R_{int} = 0.0384]
Data / restraints /parameters	5576 / 1 / 191
Goodness on fit on F^2	1.140
R_1 ($I > 2\sigma(I)$)	0.0337
wR_2 ($I > 2\sigma(I)$)	0.0990
R_1 (*all data*)	0.0345
wR_2 (all data)	0.0998
Largest diff. peak and hole, e Å^{-3}	1.65 / -0.50

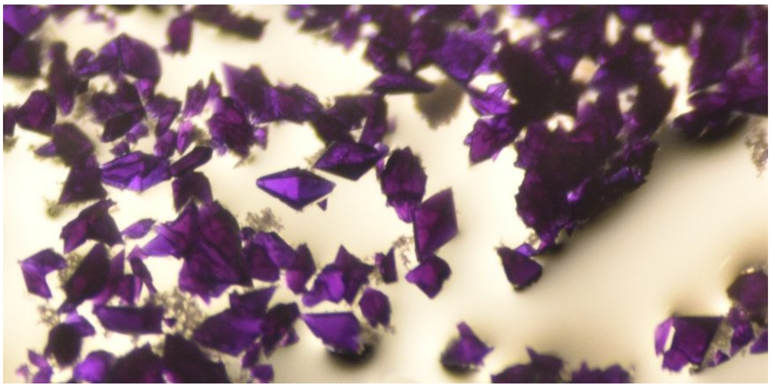

Figure S1. Photograph of the crystals of UTSA-16 employed for the structural study.

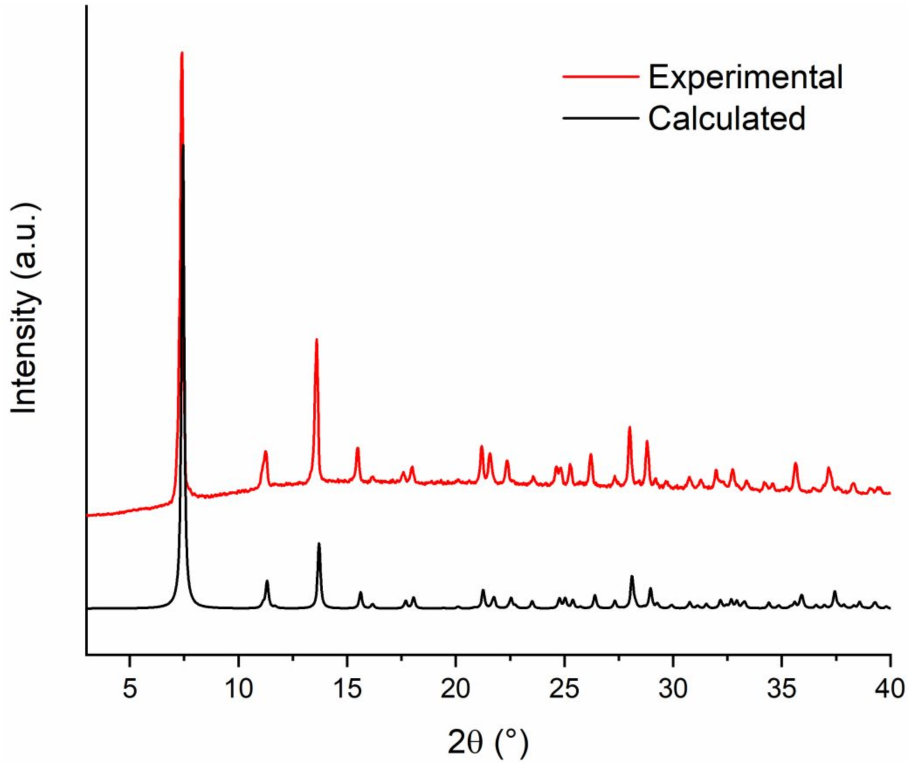

Figure S2. Comparison of the calculated PXRD pattern of UTSA-16 (black) with the experimental one (red), confirming phase purity of the sample used for chemical analysis.

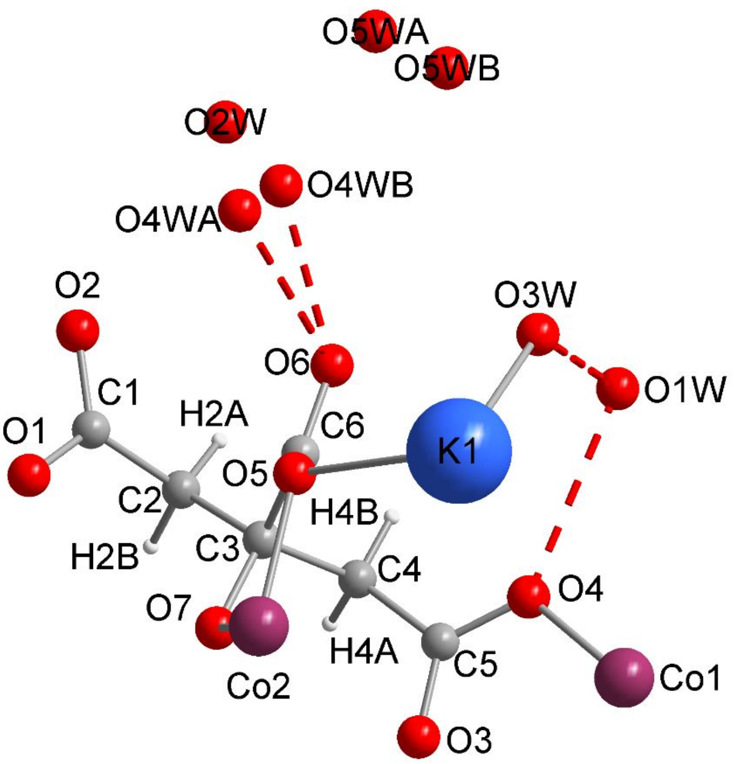

Figure S3. Asymmetric unit of RAZXIA. Colour code: Co, plum; K1, blue; C, grey; O, red; H white.

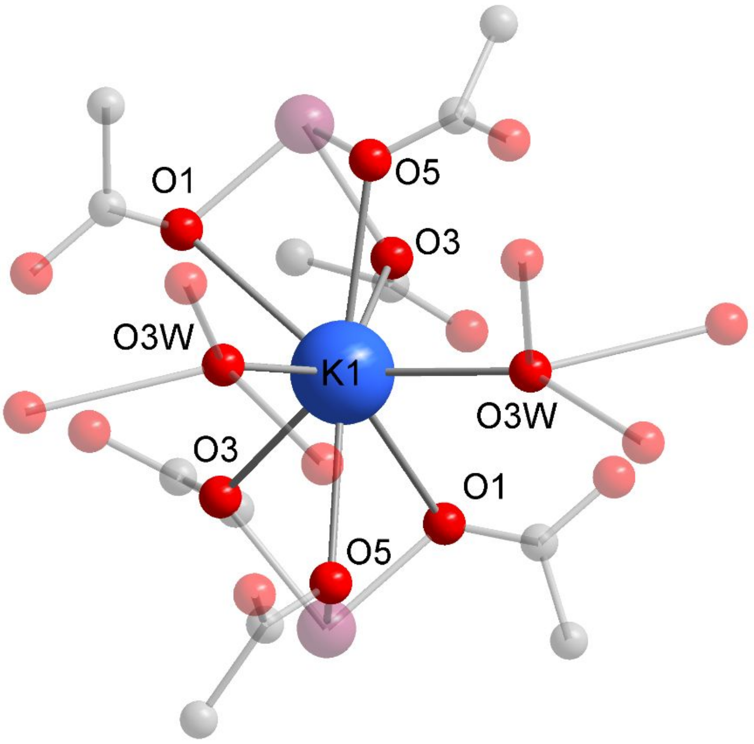

Figure S4. Environment of K1 in RAZXIA. Atoms not directly interacting with K are shaded for the sake of clarity. Colour code: Co, plum; K, blue; C, grey; O, red; H white.

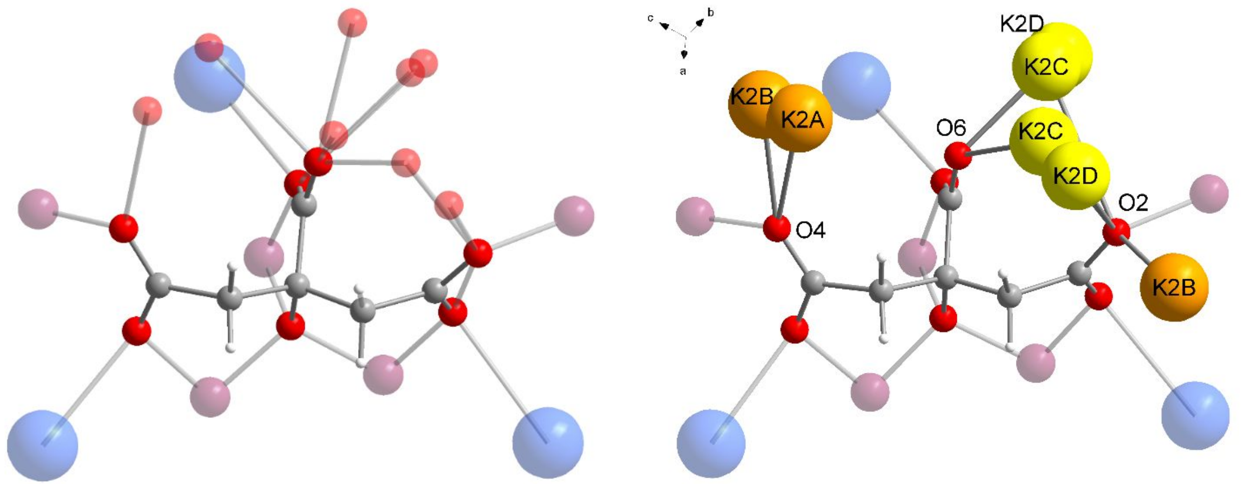

Figure S5. Environment of the linker in RAZXIA (left) and in the updated crystal structure (right). Atoms not belonging to the linker are shaded for the sake of clarity. Colour code: Co, plum; K1, blue; K2A-B, orange; K2C-D, yellow; C, grey; O, red; H white.

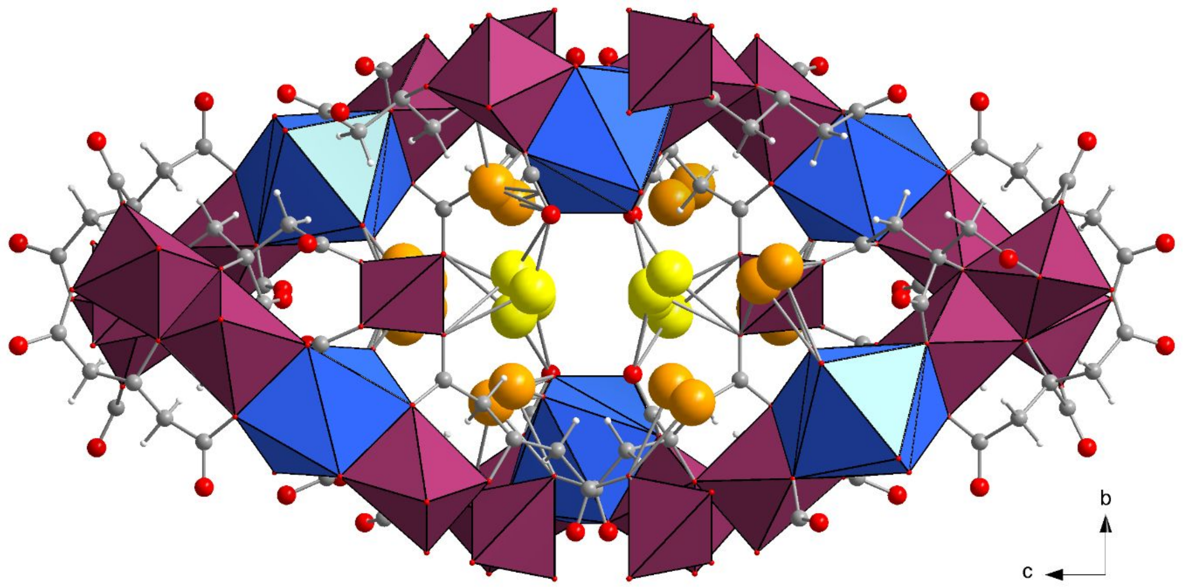

Figure S6. Polyhedral model of the updated crystal structure of UTSA-16(Co) viewed along the *a* axis. Colour code: Co, plum; K1, blue; K2A-B, orange; K2C-D, yellow; C, grey; O, red; H white.

References

(1) Xiang, S.; Wu, X.; Zhang, J.; Fu, R.; Hu, S.; Zhang, X. A 3D Canted Antiferromagnetic Porous Metal−Organic Framework with Anatase Topology through Assembly of an Analogue of Polyoxometalate. *J. Am. Chem. Soc.* **2005**, *127* (47), 16352–16353. https://doi.org/10.1021/ja0546065.

(2) Bruker AXS Inc. Bruker, APEX4 V2021.10-0, Bruker AXS Inc., Madison, Wisconsin, USA, 2021. **2021**.

(3) Bruker. SAINT v8.30A; Bruker AXS Inc.: Madison, Wisconsin, USA, 2012.

(4) Bruker AXS Inc. Bruker, XPREP V2014/2, Bruker AXS Inc., Madison, Wisconsin, USA, 2014. **2014**.

(5) Bruker AXS Inc. Bruker, SADABS V2016/2, Bruker AXS Inc., Madison, Wisconsin, USA, 2016. **2016**.

(6) Sheldrick, G. M. *SHELXT* – Integrated Space-Group and Crystal-Structure Determination. *Acta Crystallogr A Found Adv* **2015**, *71* (1), 3–8. https://doi.org/10.1107/S2053273314026370.
